# P2X7 Receptor is Involved in Mitochondrial Dysfunction Induced by Extracellular Alpha Synuclein in Neuroblastoma SH-SY5Y Cells

**DOI:** 10.3390/ijms21113959

**Published:** 2020-05-31

**Authors:** Anna Wilkaniec, Magdalena Cieślik, Emilia Murawska, Lidia Babiec, Magdalena Gąssowska-Dobrowolska, Ewelina Pałasz, Henryk Jęśko, Agata Adamczyk

**Affiliations:** 1 Department of Cellular Signalling, Mossakowski Medical Research Centre, Polish Academy of Sciences Pawińskiego 5, 02-106 Warsaw, Poland; mcieslik@imdik.pan.pl (M.C.); lbabiec@imdik.pan.pl (L.B.); mgassowska@imdik.pan.pl (M.G.-D.); epalasz@imdik.pan.pl (E.P.); hjesko@imdik.pan.pl (H.J.); aadamczyk@imdik.pan.pl (A.A.); 2 Department of Applied Microbiology, Institute of Microbiology, Warsaw University, Miecznikowa 1 Street, 02-096 Warsaw, Poland; emiliamurawska@wp.pl

**Keywords:** α-synuclein, P2X7 receptor, mitochondria dysfunction, parkin, AMP-activated protein kinase

## Abstract

The purinergic P2X7 receptor (P2X7R) belongs to a family of trimeric ion channels that are gated by extracellular adenosine 5′-triphosphate (ATP). Several studies have pointed to a role of P2X7R-dependent signalling in Parkinson's disease (PD)-related neurodegeneration. The pathology of (PD) is characterized by the formation of insoluble alpha-synuclein (α-Syn) aggregates—Lewy bodies, but the mechanisms underlying α-Syn-induced dopaminergic cell death are still partially unclear. Our previous studies indicate that extracellular α-Syn directly interact with neuronal P2X7R and induces intracellular free calcium mobilization in neuronal cells. The main objective of this study was to examine the involvement of P2X7R receptor in α-Syn-induced mitochondrial dysfunction and cell death. We found that P2X7R stimulation is responsible for α-Syn-induced oxidative stress and activation of the molecular pathways of programmed cell death. Exogenous α-Syn treatment led to P2X7R-dependent decrease in mitochondrial membrane potential as well as elevation of mitochondrial ROS production resulting in breakdown of cellular energy production. Moreover, P2X7R-dependent deregulation of AMP-activated protein kinase as well as decrease in parkin protein level could be responsible for α-Syn-induced mitophagy impairment and accumulation of dysfunctional mitochondria. P2X7R might be putative pharmacological targets in molecular mechanism of extracellular α-Syn toxicity.

## 1. Introduction

Since adenosine 5′-triphosphate (ATP) was proposed as an extracellular signalling molecule with neurotransmitter properties, the function of the purinergic signalling has been thoroughly studied in the central nervous system (CNS). Recent data highlight the involvement of purinergic neurotransmission in the pathogenesis and progression of nervous system disorders including neurodegenerative conditions, such as Alzheimer's (AD) and Parkinson's (PD) diseases [1,2,3]. The contribution of purinergic signalling is complex and involves the combined activity resulting from ATP release, its hydrolysis via ectoenzymes, and receptor activation. Two functional subclasses of membrane-bound P2 purinergic receptors, P2X(1-7) ionotropic receptors, activated by ATP, and G protein-coupled metabotropic P2Y(1-2,4,6,11-14) receptors, activated by ATP, adenosine diphosphate (ADP), and uridine di- and triphosphate (UDP and UTP), mediate the extracellular actions of ATP [4,5,6]. Most studies of the extracellular actions of ATP connected with the short-term neurotransmission and neuromodulation events are related to P2X receptor-mediated Ca^2+^ permeability and membrane depolarization. The activation of these ionotropic receptors is significant for Ca^2+^-induced intracellular signalling pathways [7,8] involved in physiological processes or pathological conditions [9]. Growing evidence shows that P2X receptors play an important role in neurodegenerative diseases [10]. Particularly, P2X7 receptor (P2X7R)-mediated signalling may exert important roles in PD-related neurodegeneration [3,11]. The P2X7R has been previously linked to a number of inflammatory diseases. However, it has only recently become evident that P2X7R also play a pivotal role in CNS pathology, because it is involved in the release of various neurotransmitters, like glutamate, GABA, and ATP from synaptic terminals, and astrocytes [12]. P2X7R-mediated Ca^2+^ influx into neuronal cells and mitochondrial dysfunction play an important role in the ATP-induced oxidative stress and neurodegeneration [13,14]. It was shown that P2X7R antagonists attenuated microglial activation and the loss of substantia nigra dopaminergic neurons in the animal models of PD [15,16,17]. Corroborating these results, we have showed that neuronal P2X7R is the important target for α-synuclein (α-Syn), the primary component of Lewy bodies and hallmark of PD [11]. A growing body of evidence suggests that α-Syn may be able to self-propagate between neurons, in a prion-like manner, which may play a pivotal role in PD pathology. Recently, it was demonstrated that the negative impact of aberrantly secreted α-Syn does not appear to involve internalization of this protein by the recipient neurons [18,19], but it depends on deregulation of various plasma membrane receptors most of which are Ca^2+^ channels [20]. Based on these data, deregulation of P2X7R-dependent purinergic signalling may be an important factor related to α-Syn-induced pathology in PD. Jiang et al. [14] showed that stimulation of the microglial P2X7 receptor by extracellular α-Syn resulted in increased oxidative stress. Consistent with these studies, we have shown that interaction of α-Syn with P2X7R is responsible for its activation, and significant [Ca^2+^]_i_ mobilization in SHSY5Y neuronal cells [11]. We showed that P2X7R/Pannexin 1 (Panx1)-dependent dynamic change of extracellular ATP and inhibition of ATP degradation are important molecular processes involved in extracellular α-Syn-mediated deleterious signalling. Since mitochondrial dysfunction was suggested as a prominent and early, chronic event that contributes to selective neuronal degeneration in PD, the main goal of this study was to investigate the role of P2X7R in extracellular α-Syn-mediated mitochondria deregulation in neuronal cells. While the significant role of purinergic P2 family receptors in neurodegenerative disorders is well known, the relationship of extracellular α-Syn with neuronal purinergic receptors as well as the involvement of this interaction on mitochondria have not yet been studied.

## 2. Results

Since extracellular α-Syn was previously shown to induce changes in P2X7R activity in neuronal cells [11], in the present study, we verified whether the deregulation of this receptor may further contribute to mitochondrial dysfunction. We used human neuroblastoma SH-SY5Y cell line, because these cells are able to express a number of features characteristic for catecholaminergic neurons, including tyrosine hydroxylase and dopamine-β-hydroxylase activities [21], as well as express various P2 receptors belonging to both P2X and P2Y families [22], including active P2X7R [11].

**Figure 1:**
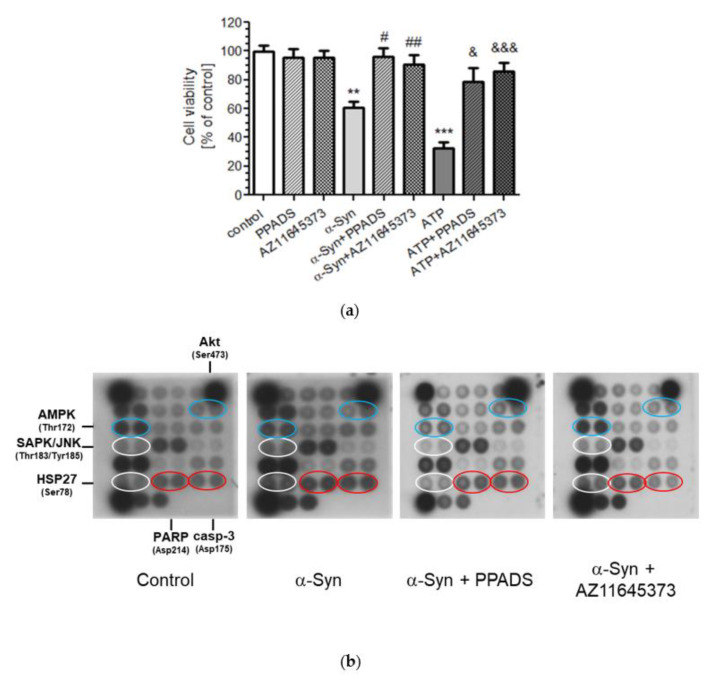

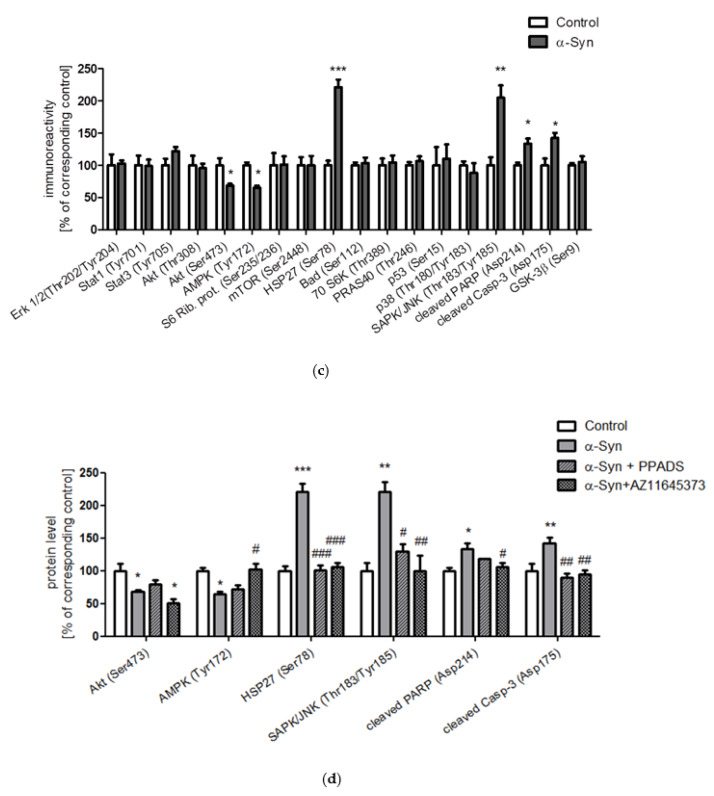
P2X7R simulation induces activation of molecular pathways of cell death in SH-SY5Y cells. (**a**) The effect of α-Syn and ATP on SH-SY5Y cells viability. SH-SY5Y cell viability after 48 h treatment with 10 μM α-Syn or 1 mM ATP in the presence of 100 μM PPADS or 10 μM AZ 11645373 measured by 2-(4,5-dimethylthiazol-2-yl)-2,5-diphenyltetrazolium bromide (MTT) test. Data represent the mean value ± S.E.M. for four independent experiments. **p < 0.01, ***p < 0.001 compared to control; #*p* < 0.05, ##*p* < 0.01 compared to α-Syn, and &*p* < 0.05, &&&*p* < 0.001 compared to ATP using one-way ANOVA followed by Bonferroni post hoc test. (**b**) PathScan® Intracellular Signaling Array Kit (Chemiluminescent Readout) was used to detect important and well-characterized signalling molecules after treatment of α-Syn (10 μM for 24 h) in the presence of 100 μM PPADS or 10 μM AZ 11645373 in SH-SY5Y cells. Images were acquired by briefly exposing the slide to standard chemiluminescent film. (**c**) Immunoreactivity of phosphorylated or truncated proteins from PathScan® Intracellular Signaling Array in SH-SY5Y cells treated with α-Syn. Data were normalized to the untreated control group (= 100%) and represent the mean value ± S.E.M. for four independent experiments. *p < 0.05, **p < 0.01; ***p < 0.001 compared to control using one-way ANOVA followed by Bonferroni post hoc test. (**d**) Immunoreactivity of selected phosphorylated or truncated proteins from PathScan® Intracellular Signaling Array in SH-SY5Y cells treated with α-Syn in the presence of 100 μM PPADS or 10 μM AZ 11645373. Data were normalized to the untreated control group (= 100%) and represent the mean value ± S.E.M. for four independent experiments. *p < 0.05, **p < 0.01; ***p < 0.001 compared to control; #*p* < 0.05, ##*p* < 0.01, ###*p* < 0.001 compared to α-Syn, using one-way ANOVA followed by Bonferroni post hoc test.

In the current study, we showed that a 48-h treatment with exogenous α-Syn (10 µM) caused significant SH-SY5Y cells death. It is previously suggested that this extracellular concentration of α-Syn is reached during pathological events that accompany the slow progression of the neurodegeneration [23,24]. To determine the contribution of P2X7R on toxicity of exogenous α-Syn, we used 100 µM PPADS, a nonselective P2 purinergic antagonist [25], or 10 µM AZ 11645373, a selective human P2X7 antagonist [26], and observed that the pretreatment with those compounds significantly prevented α-Syn-induced cell death. Similarly, the high concentration of ATP (1 mM), able to activate P2X7R, evoked neuronal cells death that was almost completely counteracted by both nonselective and selective P2X7R antagonists (Figure 1a). To further study the possible intracellular pathways responsible for α-Syn toxicity, a PathScan Intracellular Signaling Array Kit was used to detect the changes of the phosphorylation or cleavage of 18 signalling molecules of the most important signal-transduction pathways (Figure 1b). As shown in Figure 1c, treatment with exogenous α-Syn-induced twofold increase in phosphorylation of proteins that are regulated by oxidative stress conditions: HSP27 (Ser78) and SAPK/JNK (Thr183/Tyr185) in SH-SY5Y cells. Moreover, the substantial increase in the level of apoptosis indicators, activated caspase-3 (cleaved at Asp175) and cleaved PARP-1 (at Asp214), was observed in SH-SY5Y cells treated with α-Syn. We also observed the less pronounced but the significant decrease in phosphorylation of Akt at Ser473 and the decrease in phosphorylation of AMP-activated protein kinase (AMPK) on Thr172. The effect of α-Syn on the rest of the signalling molecules was negligible (Figure 1c). We further analysed the effect of nonselective and selective P2X7R antagonists on the changes in signalling pathways induced by α-Syn and observed that pretreatment with PPADS and AZ11645373 prevented HSP27 and SAPK/JNK phosphorylation, as well as caspase-3 cleavage, while only selective P2X7R antagonists prevented AMPK phosphorylation and cleavage of PARP. We also excluded the effect of P2X7R on Akt phosphorylation since both antagonists did not reverse the effects of α-Syn on post-translational modifications of this protein (Figure 1d).

**Figure 2:**
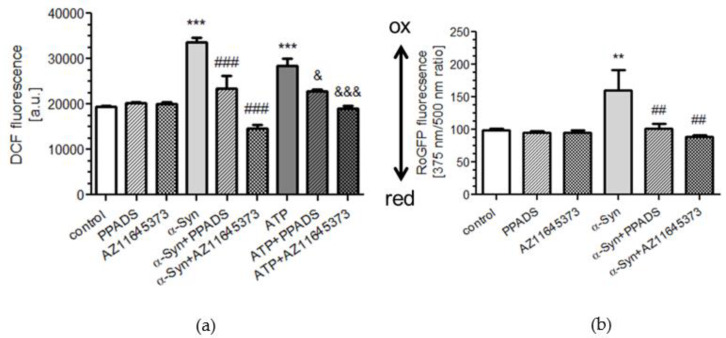
The involvement of P2X7R in oxidative stress generation in SH-SY5Y cells treated with exogenous α-Syn. (**a**) Intracellular free radicals level in SH-SY5Y cells after 8 h incubation with 10 μM α-Syn or 1 mM ATP in the presence of 100 μM PPADS or 10 μM AZ 11645373 (measured by DCF fluorescence. Data represent the mean value ± S.E.M. for four independent experiments. ***p < 0.001 compared to control, ###*p* < 0.001 compared to α-Syn, and &*p* < 0.05, &&&*p* < 0.001 compared to ATP using one-way ANOVA followed by Bonferroni post hoc test. (**b**) Oxidative-reduction potential in SH-SY5Y cells after 8 h incubation with 10 μM α-Syn in the presence of 100 μM PPADS or 10 μM AZ 11645373 measured by RoGFP fluorescence. Data represent the mean value ± S.E.M. for five independent experiments. **p < 0.01 compared to control and ##*p* < 0.01 compared to α-Syn, using one-way ANOVA followed by Bonferroni post hoc test.

Since the activation of P2X7R induced the increased phosphorylation of oxidative stress-related proteins, it is thus possible that the mechanisms of α-Syn neurotoxicity are related to the enhancement of the reactive oxygen species (ROS) level [27]. In addition, P2X7R activation induces ROS generation in various cells including neurons [28,29]. In agreement with these earlier findings, we observed that cytosolic ROS level assayed by the DCF method was significantly elevated in SH-SY5Y cells treated with extracellular α-Syn or ATP for 24 h as compared to control cells (Figure 2a). Moreover, α-Syn and ATP-induced free radicals generation was significantly prevented by pretreatment with nonselective and selective P2X7R antagonists. To verify that the cytosolic redox environment was affected by the increase in ROS, SH-SY5Y cells were transiently transfected with a reporter gene coding for a redox-sensitive green fluorescent protein (roGFP, [27]) and treated with α-Syn for 24 h. The results indicated that α-Syn significantly deregulates cellular redox state in SH-SY5Y cells. Moreover, this effect by α-Syn was significantly ameliorated by PPADS and AZ 11645373 pretreatment (Figure 2b).

**Figure 3:**
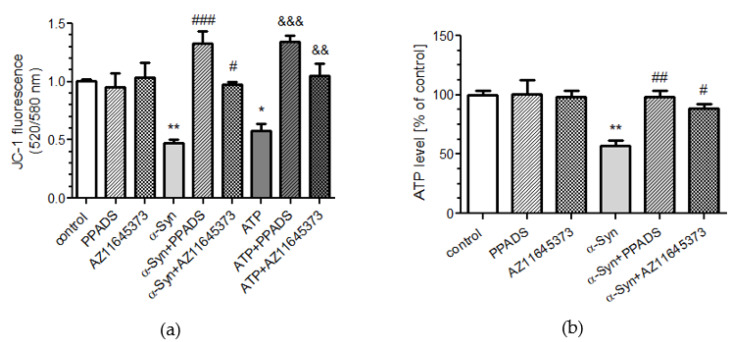
Mitochondrial dysfunction in α-Syn-treated SH-SY5Y cells is mediated by P2X7R activation. (**a**) Mitochondrial membrane potential (Δψm) was measured after 10 μM α-Syn or 1 mM ATP treatment for 8 h in the presence of 100 μM PPADS or 10 μM AZ 11645373 in SH-SY5Y cells. Data represent the mean value ± S.E.M. for four independent experiments. *p < 0.05; **p < 0.01 compared to control; #*p* < 0.05; ###*p* < 0.001 compared to α-Syn, and &&*p* < 0.01; and &&&*p* < 0.001 compared to ATP using one-way ANOVA followed by Bonferroni post hoc test. (**b**) ATP levels was measured after 10 μM α-Syn treatment for 8 h in the presence of 100 μM PPADS or 10 μM AZ 11645373 in SH-SY5Y cells. Data represent the mean value ± S.E.M. for four independent experiments. **p < 0.01 compared to control; #p < 0.05, ##*p* < 0.01, compared to α-Syn, using one-way ANOVA followed by Bonferroni post hoc test.

Our previous findings indicated that elevated oxidative stress is related to α-Syn-induced mitochondrial dysfunction [30]. To determine the involvement of P2X7R in changes of mitochondrial bioenergetics induced by acute treatment of α-Syn, the mitochondrial parameters such as mitochondrial membrane potential (MMP; indicator of polarization state of the mitochondrial membrane) and ATP level were measured in SH-SY5Y cells. We observed that a 8 h treatment with exogenous α-Syn results in a significant decrease in MMP (Figure 3a), which was paralleled by a 40% decrease of ATP level (Figure 3b), whereas pretreatment with PPADS or AZ11645373 significantly alleviates α-Syn-induced depolarization of mitochondrial membrane potential and the decrease in ATP level (Figure 3a,b). Similarly to α-Syn, treatment with ATP induced significant decrease in MMP, which was completely reversed by the nonselective and selective P2X7R antagonists (Figure 3a).

**Figure 4:**
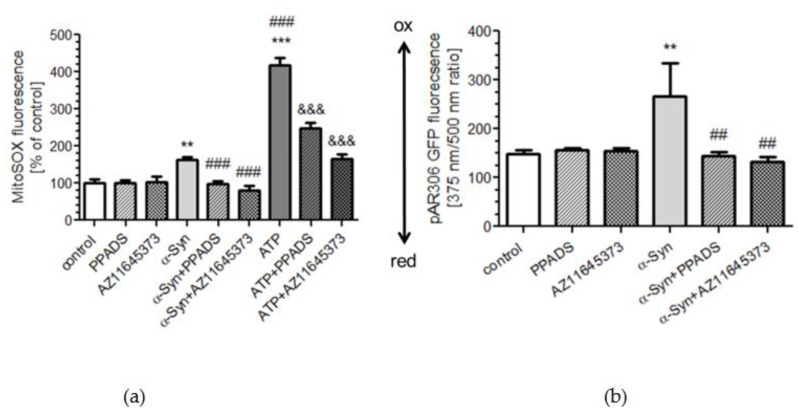
P2X7R activation modulates the mitochondrial redox environment in SH-SY5Y cells after α-Syn treatment. (**a**) Mitochondrial reactive oxygen species levels were measured using mitochondrial superoxide indicator (MitoSOX) after 10 μM α-Syn or 1 mM ATP treatment for 8 h in the presence of 100 μM PPADS or 10 μM AZ 11645373 in SH-SY5Y cells. Data represent the mean value ± S.E.M. for four independent experiments. **p < 0.01, ***p < 0.001 compared to control; ###*p* < 0.001 compared to α-Syn, and &&&*p* < 0.001 compared to ATP using one-way ANOVA followed by Bonferroni post hoc test. (**b**) Using a reporter gene coding for a redox-sensitive green fluorescent protein (pRA306 roGFP) located within mitochondria, the mitochondrial redox state was measured in SH-SY5Y cells after 8 h incubation with 10 μM α-Syn in the presence of 100 μM PPADS or 10 μM AZ 11645373 measured by RoGFP fluorescence. Data represent the mean value ± S.E.M. for four independent experiments. **p < 0.01 compared to control, ##*p* < 0.01 compared to α-Syn, using one-way ANOVA followed by Bonferroni post hoc test.

We next evaluated the free radical level within mitochondria (mtROS) and mitochondrial redox state as oxidative stress readout in SH-SY5Y cells in the presence or absence of α-Syn. Using mitochondrial superoxide indicator, MitoSOX™ Red, we observed that treatment with α-Syn results in significant elevation of mtROS, whereas in cells pretreated with P2X7R antagonists, the levels of superoxide anion were markedly reduced after α-Syn treatment (Figure 4a). In addition, mtROS levels were also enhanced in ATP-treated cells in a manner that was significantly greater than the level observed for α-Syn. Moreover, mtROS elevation induced by ATP was only partly reversed by PPADS and AZ11645373 pretreatment (Figure 4a). Finally, to verify whether the α-Syn-dependent deregulation of P2X7R affects the mitochondrial redox environment, the cells were transfected with a reporter gene coding for a redox-sensitive green fluorescent protein located within mitochondria (pRA306 GFP, Figure 4b). It was observed that 24 h treatment with exogenous α-Syn in SH-SY5Y cells results in significant increase of oxidative stress in mitochondria and P2X7R antagonists pretreatment significantly prevents α-Syn-induced changes of the mitochondrial redox state (Figure 4b). Taken together, our data indicate that exogenous α-Syn decreases mitochondrial activity, which was paralleled by decrease in ATP synthesis and elevation of mitochondrial oxidative stress, in a manner that was reversed by P2X7R blockade.

**Figure 5:**
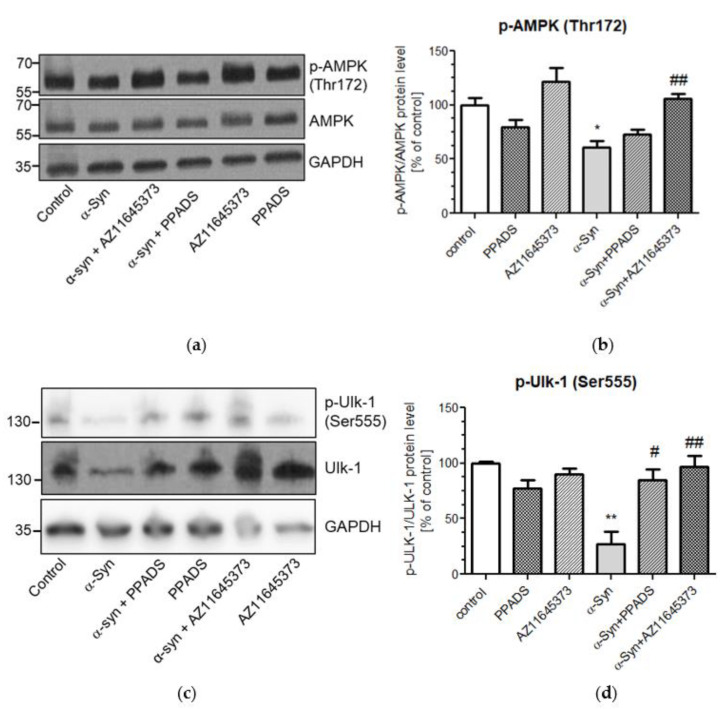
P2X7R activation induced by α-Syn treatment decreases AMPK phosphorylation in SH-SY5Y cells. (**a**) SH-SY5Y cells were treated with 10 μM α-Syn for 24 h in the presence of 100 μM PPADS or 10 μM AZ 11645373. Protein levels of phosphorylated AMPK (Thr172) and AMPK were then analysed by Western blotting. GAPDH was used as the loading control. (**b**) Phospho-AMPK immunoreactivity normalized to AMPK in SH-SY5Ycells. Data were normalized to the untreated control group (= 100%) and represent the mean value ± S.E.M. for five independent experiments. *p < 0.05 compared to control, ##*p* < 0.01 compared to α-Syn, using one-way ANOVA followed by Bonferroni post hoc test. (**c**) SH-SY5Y cells were treated with 10 μM α-Syn for 24 h in the presence of 100 μM PPADS or 10 μM AZ 11645373. Protein levels of phosphorylated Ulk-1 (Ser555) and Ulk-1 were then analysed by Western blotting. GAPDH was used as the loading control. (**d**) Phospho-Ulk-1 immunoreactivity normalized to Ulk-1 in SH-SY5Y cells. Data were normalized to the untreated control group (= 100%) and represent the mean value ± S.E.M. for four independent experiments. **p < 0.01 compared to control, #*p* < 0.05; ##*p* < 0.01 compared to α-Syn, using one-way ANOVA followed by Bonferroni post hoc test.

It is well known that mitochondria dysfunction and the decrease in intracellular ATP levels lead to activation of AMPK that is a key regulator of cellular energy metabolism. AMPK is activated by phosphorylation of α subunit at Thr172 [31], which is regulated by cellular AMP/ATP ratio, Ca^2+^ concentration, and ROS [32,33]. Therefore, we investigated AMPK activation by analysis of the level of its phosphorylation at Thr172. Similarly, as in Path Scan assay, the Western blot analysis showed that treatment with exogenous α-Syn does not change the protein level of AMPK, but it significantly decreases AMPK phosphorylation (Figure 5a). Moreover, the effect of α-Syn was reversed exclusively by selective P2X7R antagonist treatment, whereas PPADS had no effect on AMPK phosphorylation induced by α-Syn (Figure 5b). Accordingly, the AMPK-dependent phosphorylation of Ulk-1 (UNC-51-like kinase 1) was also decreased in cells treated with exogenous α-Syn. (Figure 5c). This effect of this protein was reversed by pretreatment with either nonselective and selective P2X7R antagonists (Figure 5d).

**Figure 6:**
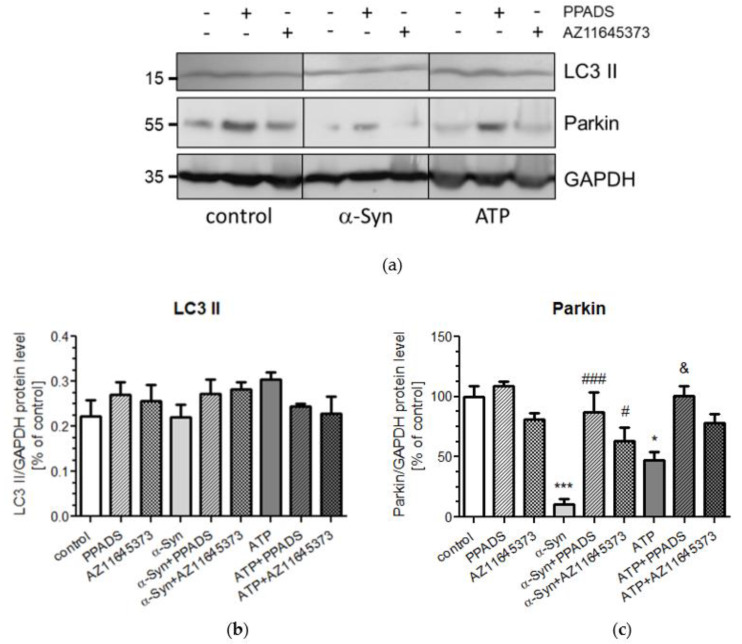
P2X7R activation induced by α-Syn treatment does not change the protein level of LC3β II but decreases parkin level in SH-SY5Y cells. (**a**) SH-SY5Y cells were treated with 10 μM α-Syn or 1 mM ATP for 24 h in the presence of 100 μM PPADS or 10 μM AZ 11645373. Protein levels of LC3β II and parkin were then analysed by Western blotting. GAPDH was used as the loading control. “+” with treatment; “-“ without treatment (**b**) LC3β II immunoreactivity normalized to GAPDH in SH-SY5Y cells. Data were normalized to the untreated control group (= 100%) and represent the mean value ± S.E.M. for three to seven independent experiments. (**c**) Parkin immunoreactivity normalized to GAPDH in SH-SY5Y cells. Data were normalized to the untreated control group (= 100%) and represent the mean value ± S.E.M. for four independent experiments. *p < 0.05; ***p < 0.001 compared to control, #*p* < 0.05; ###*p* < 0.001 compared to α-Syn, and &*p* < 0.05 compared to ATP using one-way ANOVA followed by Bonferroni post hoc test.

To further investigate the effect of α-Syn on general autophagy process, the level of the microtubule-associated protein 1A light chain 3 II (LC3-II), a form of LC3-phosphatidylethanolamine conjugate, which is recruited to autophagosomal membranes and serve as marker of autophagy [34], was investigated by Western blot analysis (Figure 6a). We observed that treatment of SH-SY5Y cells with exogenous α-Syn does not have a significant impact on the LC3II formation (Figure 6b). Since efficient autophagic degradation of mitochondria (mitophagy) requires the participation of parkin, the level of this protein was measured by Western blot analysis (Figure 6a). We observed that α-Syn treatment induced the significant decrease of parkin levels in SH-SY5Y cells, whereas nonselective and selective P2X7R antagonists normalized parkin level in cells treated with α-Syn. Similarly, we observed that exogenous ATP has significant but less pronounced effect on parkin protein level, and this effect was reversed only by nonselective P2X7R antagonist (Figure 6c). 

**Figure 7:**
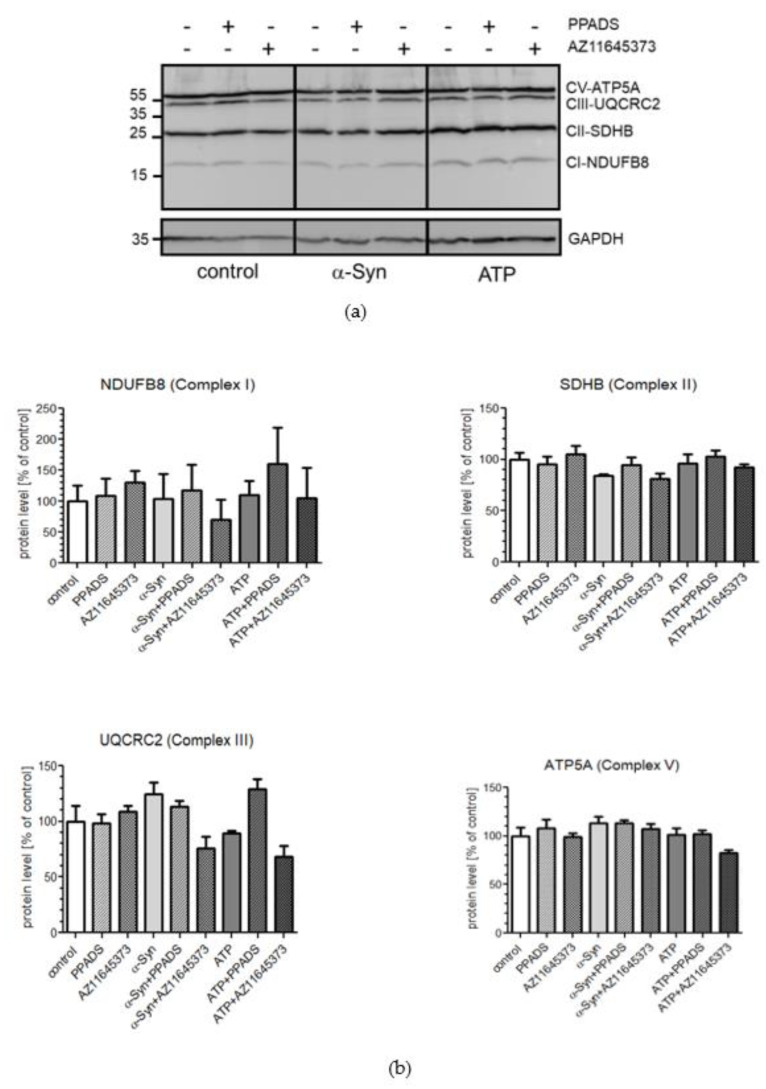
P2X7R activation induced by α-Syn treatment does not change the mitochondrial proteins level. (**a**) SH-SY5Y cells were treated with 10 μM α-Syn or 1 mM ATP for 24 h in the presence of 100 μM PPADS or 10 μM AZ 11645373. Protein levels of representative mitochondrial markers were analysed by Western blotting. GAPDH was used as the loading control. “+” with treatment; “-“ without treatment (**b**) Mitochondrial markers immunoreactivity normalized to GAPDH in SH-SY5Y cells. Data were normalized to the untreated control group (= 100%) and represent the mean value ± S.E.M. for four independent experiments.

In order to characterize mitochondria level following acute α-Syn treatment, we performed Western blot analysis of a representative subunit from each of the five OXPHOS complexes, but we were unable to consistently detect the protein band for complex IV in our samples (Figure 7a). Results showed that the protein levels of complexes I, II, III, and V remain unchanged upon P2X7R stimulation by α-Syn or ATP, i.e., no significant difference in protein levels was detected between treated and control cells (Figure 7b).

## 3. Discussion

Recent data indicated that P2X7 receptor is a molecular target of extracellular α-Syn in both neuronal and microglial cells and that the interaction of α-Syn with P2X7R is responsible for its activation [11,14]. Moreover, a few studies demonstrate that oxidative stress and mitochondrial toxicity are the key events leading to cell death induced by P2X7R [13,35]. In line with those data, we showed for the first time that exogenous α-Syn leads to P2X7R-dependent deregulation of mitochondria function resulting in decrease in cellular energy production and cell death.

Many studies indicated deregulation in calcium homeostasis and elevated release of free radicals as important mediators of toxicity induced by extracellular α-Syn [20,27]. Among various receptors through which extracellular α-Syn induces calcium influx, P2X7R is one of the most potent inductors of oxidative stress and cell death. It was previously demonstrated that direct association of α-Syn with P2X7R leads to rapid increase in intracellular calcium load that was mainly associated with Ca^2+^ influx through formation of P2X7R/Panx 1 pore [11]. Persistent activation of P2X7R by high ATP concentrations leads to death of various cell types within CNS, including macrophages, microglia, and neurons, by the mechanisms that are closely connected with Ca^2+^ overload and free radicals production [13,29,35,36]. However, over the past years, the major scientific interest was focused mainly on the potential role of P2X7 in microglial activity, where P2X7 is ubiquitously expressed [37,38,39,40,41,42,43]. P2X7R in microglia has been considered as a drug target for CNS disorders such as ischaemia [44,45], traumatic brain injury [46], spinal cord injury [47], epilepsy [48], Alzheimer's disease [49,50], Parkinson’s disease [15], prion disease [51], or Huntington's disease [52]. It was due to the fact that ATP is one of the recognizing damage-associated molecular patterns (DAMPs), therefore microglial P2X7R acts as a pattern recognition receptor, which is activated by high concentration of extracellular ATP released from dying cells due to brain injuries or neurodegeneration [53]. Activation of the microglial P2X7R initiates innate immunity by promoting assembly of the caspase-1-activating platform known as the NLRP3 inflammasome [54,55]. Moreover, P2X7R was shown to be an obligate participant in microglia activation caused by amyloid beta and α-Syn [14,56,57]. However, recent data also suggest that P2X7R is functionally expressed in neuronal cells and its activation has a direct impact on neurodegeneration [13,15,58,59]. In line with those studies, our data indicated that activation of P2X7R by extracellular α-Syn is responsible for the significant decrease in viability of neuronal cells. Until now, the involvement of P2X7R in various cell death pathways, including apoptosis, pyroptosis, necrosis, and autophagy was shown, depending upon the incubation time, agonist dose, and cell type [60]. It was previously demonstrated that high ATP concentrations stimulated a necrotic cell death that was characterized by early cell swelling and cell lysis [61,62]. Conversely, P2X7R activation was also shown to regulate apoptotic cell death via ROS-dependent cytochrome c release and caspase-3/7 activation [13,63,64]. In line with those observations, our study demonstrated that stimulation of P2X7R with α-Syn induced oxidative stress, mitochondria dysfunction, and caspase-3 cleavage. Moreover, P2X7R activation by exogenous α-Syn led to stimulation of JNK pathway that has been identified as a key element responsible for the regulation of apoptosis signals and critical for cell death associated with neurodegenerative diseases [65]. Upon α-Syn-mediated stimulation of P2X7R, the increased activation of HSP27 was also observed. HSP27 is a stress response protein; its phosphorylation shows an increased level several minutes after exposure to stress and returns to basal levels after removal of the stress events [66]. Although the activation of HSP27 is believed to be one of the mechanisms of cellular prevention against apoptosis, mainly through inhibition of caspases [67,68], its predominant function is inhibiting the oxidative stress, by modulating and maintaining the redox parameters, especially glutathione levels within the cells [69,70]. However, our study demonstrated that although the significant stimulation of HSP27 occurs, this protein was unable to prevent deregulation of the redox homeostasis upon P2X7R activation by α-Syn.

Interestingly, we observed the higher toxicity of exogenous ATP than the toxicity evoked by α-Syn, suggesting that stimulatory effect of α-Syn on P2X7R may vary from the one exerted by ATP. That raises the question about the nature of α-Syn and P2X7R interaction. It was previously demonstrated that P2X7R co-immunoprecipitates with α-Syn [14]. In the conditions of extracellular ATP withdrawal, the exogenous α-Syn was still able to activate P2X7R and this effect is reversed by selective inhibitor of P2X7R [11]. Based on those data, it could be speculated that exogenous α-Syn can directly activate P2X7R, however, the site of interaction of this protein with P2X7R subunits remains unknown. Although it could not be excluded that the primary location of α-Syn binding is the agonist site, it is also possible that interaction between α-Syn and P2X7R occurs at transmembrane regions, as α-Syn contain amino acids that promote membrane insertion. This interaction might induce conformational changes of P2X7R leading to its activation. Especially, the possibility of the interaction between α-Syn and large C-terminus of P2X7R seems to be very interesting. This domain of P2X7R is the longest among the whole subfamily of P2X receptors and is responsible for the unique properties of this receptor [71]. The C-terminus of the P2X7R has been implicated in regulating receptor function including signalling pathway activation, cellular localization, protein–protein interactions, and post-translational modifications [72,73,74]. Our previous study indicated that activation of P2X7R by exogenous α-Syn leads to the recruitment of Panx1 [11], which is suggested to be the real pore opening controlled by P2X7R for the transport of ions [75,76]. Since it is evidenced that the Src homology 3 death domain (SH3) of the C-terminus of the P2X7R is involved in the initial steps of the signal transduction events leading to Panx1 activation [77,78], it is highly probable that this is also a primary site of α-Syn–P2X7R interaction. Moreover, since we observed the negligible effect of α-Syn on ERK 1/2 activation that was shown to depend mainly on the N-terminus of P2X7R [79], this might be another prerequisite confirming the assumption of interaction between α-Syn and C-terminus of P2X7R. However, this interesting hypothesis needs to be further elucidated. 

It was previously evidenced that oxidative stress and mitochondria play an important role in stimulating apoptosis, while mitochondria are believed to be both a target and source of ROS [80]. ROS initiate the mitochondria-dependent intrinsic pathway of apoptosis and promote the activation of proapoptotic proteins [81]. There is growing evidence that the mitochondrial damage followed by activation of mitochondrial pathway of apoptosis are the major cause of neurodegeneration evoked by α-Syn [20,30,82,83]. It was observed that mitochondrial membrane potential and ATP production were either affected upon exogenous administration of the recombinant wild-type and mutant α-Syn [84] or by overexpression of wild-type or mutated α-Syn [82,85]. The decline in mitochondrial respiration through mitochondrial depolarization and disturbances in mitochondrial complex I activity followed by an elevation in free radicals production are believed to be the key molecular events activated by α-Syn [86,87,88]. In line with those studies, our results showed that extracellular α-Syn induces mitochondrial depolarization followed by elevation of mitochondrial superoxide level as well as deregulation of mitochondrial redox homeostasis. Moreover, this deleterious effect of α-Syn is largely dependent on activation of P2X7R. Previous data suggested that the translocation of either wild-type or mutant α-Syn to mitochondria [89,90] followed by the direct interaction of α-Syn with mitochondria-associated endoplasmic reticulum membranes (MAMs) [91,92] are the major mechanisms of α-Syn-induced mitochondrial dysfunction and elevation of free radicals production [88,93]. In light of these data, the involvement of P2X7R in those mechanisms are elusive. Since the activation of P2X7R by extracellular α-Syn is previously shown to induce the recruitment of Panx-1 [11] and the formation of pore, permeable to large molecules of up to 900 Da in size, it is thus possible that toxic effect of extracellular α-Syn is mediated by internalization of this protein through P2X7R. Yet, this interesting hypothesis requires further investigation. It is also possible that α-Syn may also induce mitochondrial dysfunction indirectly by generating a P2X7R-dependent calcium and nitric oxide increase with consequent nitrosylation of mitochondrial proteins [30,93,94]. It was previously reported that P2X7R-triggered Ca^2+^ entry [13] and the formation of ROS [35,39] are responsible for the decline in mitochondrial respiration and activation of the apoptosis cascade in neuronal cells [95]. According to those reports, oxidative modifications of mitochondrial components induced by α-Syn were shown to be responsible for release of cytochrome c from mitochondria, activation of caspases cascade, and neuronal cells death [82,96]. Recently, the involvement of ER that form structural and functional networks with mitochondria, in the mitochondrial membrane permeabilization and apoptosis under various pathophysiological conditions, was suggested [97]. Moreover, it was previously demonstrated that ER stress is involved in P2X7R-mediated neurotoxicity in neuronal cells in the manner that is largely dependent from sustained Ca^2+^ depletion from ER stores [98]. Since Ca^2+^ released from ER is taken up by mitochondria that results in calcium overload and induces depolarization of mitochondrial membrane and opening of permeability transition pore (PTP) to release apoptotic proteins able to stimulate caspase cascade [99], it is though possible that P2X7R-mediated Ca^2+^ release from ER might be responsible for mitochondria dysfunction. Although our previous studies showed that rapid elevation of [Ca^2+^]_i_ after α-Syn treatment is not related to ER stores mobilization upon P2X7R stimulation [11], the involvement of ER in Ca^2+^ deregulation in later time points leading to mitochondrial dysfunction cannot be ruled out entirely. Remarkably, we observed that generation of mtROS upon stimulation with exogenous ATP was almost twofold higher than this induced by α-Syn. It was previously demonstrated that elevated levels of intracellular Ca^2+^ were critical in the generation of mitochondrial but not cellular ROS following treatment with ATP [61]. Taking these observations into consideration, it might be possible that the mechanisms of α-Syn-induced P2X7R-mediated mitochondria dysfunction might be not entirely dependent on Ca^2+^ overload. Again, those data might suggest the involvement of SH3 death domain within C-terminus of P2X7R in the toxicity evoked by exogenous α-Syn. 

A plethora of studies demonstrate that mitochondrial damage and failure in ATP synthesis are activators of AMPK protein complex, which is a central regulator of cellular energy homeostasis and survival [100,101]. AMPK is also crucial for mediating mitophagy and modulating mitochondrial dynamics and biogenesis [102]. AMPK and a downstream regulator of autophagy/mitophagy, Ulk1, have been shown to play critical roles in mitophagy in neuronal cells [103,104]. Alterations of AMPK signalling have been shown in several brain disease models, including PD [105]. However, the neuronal effects of AMPK activation are not fully elucidated and are controversial, as it is demonstrated that stimulation of this kinase may be either protective or detrimental [106,107,108,109,110]. Nevertheless, in all PD models based on neurotoxins that disrupt the activity of mitochondrial complex I, the sustained activation of AMPK was observed. Since α-Syn may severely impair complex I, it could also promote the increase in AMPK levels through a compensative autoregulatory mechanism. However, in our study, we observed the inhibition of AMPK phosphorylation on Thr172, suggesting the decrease in this protein activity. Although the inhibitory effect of exogenous α-Syn might seem surprising, these observations are in agreement with the previous studies showing that both α-Syn overexpression and extracellular treatment downregulate AMPK activation and that restoration of AMPK activity reduces the neurotoxicity of α-Syn in vitro [111]. Moreover, it was evidenced that overexpression of the AMPK can protect neurons at early stages of the α-Syn pathology, in a manner that was attributed to the restoration of deregulated autophagy and mitophagy [112]. Taken together, those data suggest that AMPK deregulation might play a significant role in the mechanisms of neurotoxicity of α-Syn. Previously, the involvement of P2X7R in modulation of AMPK activity and regulation of autophagic flux was highlighted. It was documented that AMPK is a key signalling modulator of P2X7R to induce mitophagy and mitochondrial fission in microglia [113] and that the deleterious effect of P2X7R predominantly include lysosomal impairment in microglial cells [113,114]. Conversely, in our study, we demonstrated that α-Syn-dependent stimulation of P2X7R in neuronal cells results in inhibition of AMPK activity, followed by the inhibition of Ulk-1, which might have the negative effect on autophagy initiation. While the opposite effect of P2X7R stimulation on AMPK activity remains to be further elucidated, it is though possible that different cell types might activate different cellular pathways upon P2X7R stimulation that is attributed to their specific function and differences in P2X7R expression [115]. Moreover, the effects of P2X7R on AMPK might be also time dependent. In the study of Sekar et al. [113], the activation of AMPK was observed shortly after P2X7R stimulation, whereas in our study, the decline in AMPK phosphorylation was observed after the prolonged P2X7R stimulation. Another issue concerns the mechanism of P2X7R-dependent decrease in AMPK phosphorylation. AMPK activation is regulated by multiple upstream signalling molecules on which, the protein phosphatase 2A (PP2A), which dephosphorylates and deactivates neuronal AMPK [116], was shown to be the molecular target of P2X7R activity [117]. PP2A has also been shown to be activated by α-Syn in neurons [118]. Therefore, it is plausible that calcium signalling activated by P2X7R stimulation might be the molecular mechanism of α-Syn-dependent decrease in AMPK activity.

Apart from the involvement of α-Syn in the regulation of the mitophagy initiation, it was demonstrated that α-Syn overexpression inhibits autophagosome synthesis resulting in accumulation of autophagy substrates [119], suggesting that the macroautophagy dysfunction may be a direct cause of abnormalities in damaged mitochondria removal. However, unlike the previous studies in cells overexpressing α-Syn, showing the significant decrease of the level of LC3-II, which is known to be a robust marker of autophagosomes [119], the recent study indicates that the expression of LC3-II is unchanged upon exogenous α-Syn treatment. Interestingly, some studies using conditional A53T transgenic mouse model show increased levels of lysosomal markers in aged DA neurons [120], while other suggest that autophagic activity is impaired only by aggregated forms of intracellular α-Syn [121]. This discrepancy might suggest that depending on aggregation, mutation, or way of administration, α-Syn might have a different impact on autophagy. 

Finally, in this study, it was observed that activation of neuronal P2X7R causes reduction of parkin protein level after exogenous α-Syn administration in dopaminergic cells. Our previous study indicated that α-Syn-induced oxidative/nitrosative stress evoked parkin post-translational modifications and degradation [27]. Given that exogenous α-Syn or high ATP concentration induces extensive liberation of ROS, it is though possible that upon P2X7 activation, oxidative modifications of parkin are responsible for downregulation of this protein. However, our study suggested that stimulation of P2X7R by α-Syn is responsible only to some extent for the degradation of parkin, since treatment with selective antagonist of P2X7R only partially prevented parkin downregulation induced by α-Syn. Moreover, the effect of ATP treatment on parkin protein level was weaker that this exerted by α-Syn. The reason for this might be related to the mechanisms of α-Syn-mediated activation of neuronal nitric oxide synthase (nNOS) activity, predominantly involved in parkin nitrosylation and downregulation [27]. While P2X7R stimulation was able to activate nNOS in hippocampal neurons in a manner that was independent of glutamate signalling [122], the stimulation of neuronal NMDA receptor was previously demonstrated to be the important mechanism of α-Syn-evoked nNOS activation [123]. Thus, the observed decrease in parkin level induced by exogenous α-Syn could be associated with the activation of either purinergic or glutamatergic signalling cascades. It is also possible that other post-translational modifications of parkin that are independent from P2X7R activation could be involved in parkin degradation induced by α-Syn. In agreement with this hypothesis, a recently published paper by Chen et al. [124] demonstrated that phosphorylation of parkin at Ser131 was responsible for disruption of the parkin’s protective function in A53T transgenic mice model of synucleinopathy. Parkin deregulation was previously demonstrated to be closely connected with mitochondria dysfunction [125]. In parkin-deficient mice, impairment in the respiratory capacity and increased protein and lipid peroxidation were observed, concomitantly with significant reduction in expression of proteins regulating mitochondrial function and antioxidative defence [126,127]. Similarly, the in vitro studies on fibroblasts isolated from PD patients showed that the decrease in parkin function due to mutations or gene silencing leads to mitochondrial depolarization, decrease in complex I activity, and ATP-production [128]. Our promising preliminary follow-up studies showed that parkin overexpression protects against the toxic effects of α-Syn, and boosting up parkin level prevents mitochondrial dysfunction induced by exogenous α-Syn (own unpublished data). Accumulation of abnormal mitochondria in PD patients with parkin mutations was suggested to be the direct cause of neurodegenerative changes [129,130,131]. Furthermore, it was demonstrated that the translocation of parkin to depolarized mitochondria initiates the process of mitophagy. Subsequently, as mitochondria-anchored parkin ubiquitinates proteins on the OMM [132,133], the recruitment of autophagic adaptor proteins to mitochondria occurs, thus facilitating their elimination by mitophagy [134,135]. Considering the negative impact of exogenous α-Syn on the protein level of parkin [27] as well as the essential role of parkin for mitochondrial quality control in a number of models [136], it is thus possible that upon α-Syn treatment, failure of parkin function is a major mechanism responsible for the persistence of damaged mitochondria. Indeed, in the present study, we found that despite extensive mitochondrial depolarization and decrease in cellular energy production, there was no significant change in the level of mitochondria upon exogenous α-Syn treatment. These results suggest that the deregulation of both AMPK activity and parkin level might induce a general breakdown of the mechanism responsible for the mitophagy that ultimately leads to accumulation of damaged mitochondria within the cell. 

**Figure 8:**
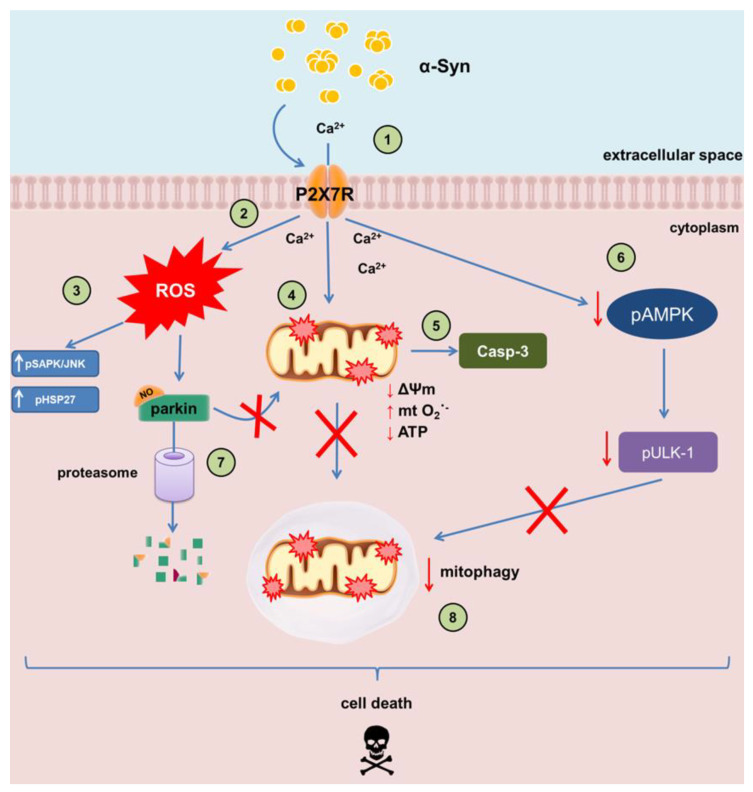
A schematic illustration of the molecular mechanisms involved in P2X7R-mediated neurotoxicity of α-Syn. (1) Extracellular α-Syn is able to bind with and activate P2X7R. (2) Activation of P2X7R leads to the increased Ca^2+^ influx and generation of ROS. (3) Elevation of free radicals synthesis leads to activation of stress response proteins SAPK/JNK and HSP27. (4) Stimulation of P2X7R results in mitochondria failure: decrease in mitochondrial membrane potential, increase in mitochondrial ROS, and decrease in ATP synthesis. (5) Mitochondrial dysfunction leads to activation of caspase-3, the major executor of mitochondria-dependent intrinsic pathway of apoptosis. (6) P2X7R activation leads to decrease in activity of AMPK, which is a central regulator of mitophagy through phosphorylation of Ulk-1. (7) ROS generated by P2X7R activation induces parkin nitrosylation and degradation in proteasome. (8) The decrease in AMPK activity and parkin downregulation might induce a general breakdown of the mechanism responsible for the mitophagy that ultimately led to accumulation of damaged mitochondria within the cell. Taken together, activation of oxidative stress, mitochondria dysfunction, and deregulation of mitophagy result in induction of neuronal cells death.

Summarizing, in this work, we provide a documentation linking functional aspects of mitochondria dysfunction to P2X7R deregulation evoked by exogenous α-Syn. Our study showed for the first time that the α-Syn-induced activation of P2X7R is responsible for ROS-mediated mitochondrial dysfunction as well as deregulation of AMPK and parkin that might result in the accumulation of defective mitochondria (Figure 8). Therefore, the obtained results might help in verification of accepted views about PD pathomechanisms and state the strong basis for the future experiments.

## 4. Materials and Methods 

### 4.1. Materials

α-Syn was obtained from rPeptide (Bogart, GA, USA). Further, 3-[1-[[(3′-nitro[1,1′-biphenyl]-4-yl)oxy]methyl]-3-(4-pyridinyl)propyl]-2,4-thiazolidinedione (AZ 11645373) was obtained from Tocris Bioscience (Bristol, UK). Neuroblastoma SH-SY5Y cell line and cell culture reagents, such as minimum essential medium eagle (MEM), Ham's F12 medium, Hank's balanced salt solution (HBSS), nonessential amino acid solution, foetal bovine serum (FBS), penicillin, streptomycin, L-glutamine, Bradford Reagent, Accutase® solution; antibodies, such as anti-glyceraldehyde 3-phosphate dehydrogenase (GAPDH), anti-rabbit IgG; and other reagents, such as pyridoxal-5′-phosphate-6-azo-phenyl-2,4-disulfonate (PPADS), dimethyl sulfoxide (DMSO), and bovine serum albumin (BSA), were purchased from Sigma-Aldrich (St. Louis, MO, USA). Clarity™ Western ECL Substrate was purchased from Bio-Rad Laboratories (Hercules, CA, USA). Complete® protease inhibitor mixture tablets were purchased from Roche Diagnostics. Cell lysis buffer and antibodies, such as rabbit antiparkin (#2132), rabbit anti-AMPK (#5832), rabbit anti-Ulk-1 (#8054), rabbit anti-p-Ulk-1 (#5869), rabbit anti-p-AMPK (#2535), rabbit anti-LC3-II (#2775), were obtained from Cell Signaling Technology (Beverly, MA, USA). Total OXPHOS Rodent WB antibody cocktail was purchased from Abcam (Cambridge, UK). BD™ MitoScreen (JC-1) was purchased from Becton Dickinson (Franklin Lakes, New Jersey, USA). MitoSOX Red and 2',7'-dichlorodihydrofluorescein diacetate (H2DCFDA) were purchased from Thermo Fisher Scientific (Waltham, Massachusetts, USA). ViaLight^TM^ Plus Kit was purchased from Lonza (Basel, Switzerland). All other reagents were purchased from POCH (Gliwice, Poland).

### 4.2. Preparation of Soluble α-Syn 

Human α-Syn was dissolved in phosphate-buffered saline (PBS) (pH 7.4) at a concentration of 100 μM and immediately used for experiments as soluble α-Syn in the form of mixture of monomers and oligomers [30].

### 4.3. Cell Culture

The studies were carried out using human neuroblastoma SH-SY5Y cell line, which is known to be able to both proliferate and differentiate in culture. SH-SY5Y cells were cultured in F12/MEM medium supplemented with 15% heat-inactivated FBS, 1% nonessential amino acids, 50 units/mL penicillin, 50 µg/mL streptomycin, and L-glutamine at 37 °C in a humidified incubator containing 5% CO_2_. 

### 4.4. Cellular Treatment 

SH-SY5Y cells were plated in 60 and 35-mm culture dishes or 96-well plates and the growth medium was changed into a low-serum medium (MEM/F12 supplemented with 2% FBS, 1% penicillin/streptomycin, and 1% L-glutamine). HBSS or other media appropriate for the particular procedure were also be used. Then, the cells were treated with exogenous α-Syn (10 μM), specific agonist and antagonists of purinergic receptors, i.e., ATP (1 mM, pH 7.3–7.5), PPADS (100 µM, dissolved in H2O), and AZ 11645373 (10 µM, dissolved in DMSO), for appropriate time points. Appropriate solvent was added to respective controls, moreover, the appropriate volume of DMSO was applied to every experimental group (control, α-Syn, ATP, etc.).

### 4.5. Cell Viability 

Cellular viability was evaluated by the reduction of 2-(4,5-dimethylthiazol-2-yl)-2,5-diphenyltetrazolium bromide (MTT) to formazan. Low-serum medium containing investigated substances were added to the cells for 48 h. MTT (2.5 mg/mL) was added to all wells and allowed to incubate at 37 °C for 2 h, followed by cell lysis and spectrophotometric measurement at 595 nm.

### 4.6. Measurement of Intracellular Free Radicals Level 

Measurement of the free radicals level was carried out using fluorescent indicator 2'7'-dichlorofluorescein diacetate (DCFH-DA), as described previously [30]. DCFH-DA is intracellularly deacetylated to 2'7'-dichlorofluorescin (DCFH) and then oxidized by hydrogen peroxide to a fluorescent compound, 2'7'-dichlorofluorescein (DCF). SH-SY5Y cells were incubated in DCFH-DA (10 µM) solution in HBSS with 20 mM HEPES (pH 7.4) and 0.02% Pluronic for 50 min at 37 °C in the dark. Then the cells were washed three times, and the DCF fluorescence was measured using a microplate reader FLUOstar Omega (Ortenberg, Germany) at 485 nm excitation and 538 nm emission wavelengths. After determining the baseline fluorescence of the cells incubated in HBSS, the changes in fluorescence after the addition of the test compounds were recorded every 1 h for 8 h. The results of fluorescence measurements are presented as percent of corresponding control. 

### 4.7. Measurement of Mitochondrial ROS Production Using MitoSOX Red

Mitochondrial superoxide production was measured using the MitoSOX Red fluorescent probe according to [137] with modifications. Cells were plated in 8 replicates into a black 96-well cell culture plate at a density of 1.5 × 10^4^ cells/well. After 24 h incubation in the presence of tested compounds, cells were washed twice with HBSS to remove the medium and subsequently incubated for 10 min (needed to allow the probe to enter the cell and start the reaction within the mitochondria) at 37 °C in 100 µL of measurement buffer containing 2.5 µM MitoSOX Red. After the incubation, the cells were washed twice with HBSS. The fluorescence was monitored in the measurement buffer with a Tecan Infinite M200 plate reader (Tecan US Inc., Durham, NC, USA) set to 510 nm excitation (Ex bandwidth: 10 nm) and 595 nm emission (Em bandwidth: 35 nm) wavelengths.

### 4.8. Cytosolic Redox Environment

To investigate changes in cytosolic redox environment, SH-SY5Y cells were transfected with a plasmid coding for a redox-sensitive green fluorescent protein (roGFP in pEGFP-N1). In an oxidized environment, the absorption increases at short wavelengths (375 nm) at the expense of absorption at longer wavelengths (500 nm). The fluorescence ratio indicates oxidation/reduction as described previously by Cannon and Remington [138]. SH-SY5Y cells were transfected using electroporation (Neon Transfection System) in 100 μL volume containing 1.4 × 10^6^ cells and 20 μg DNA, at manufacturer’s SH-SY5Y-optimized pulse parameters (Thermo Fisher Scientific). Cells were plated in 4 replicates onto 96-well plates at a density of 1.5 × 104 cells/well in standard culture medium less antibiotics and kept overnight at 37 °C in 5% CO_2_. After 24 h treatment with α-Syn, cells were washed twice with PBS and placed in a Hank’s buffer. The ratio 375 nm/500 nm was measured using multiplate reader Infinite M1000 PRO (TECAN). An increase of the ratio indicates a more oxidized environment. 

### 4.9. Determination of Mitochondrial Membrane Potential

The mitochondrial membrane potential in SH-SY5Y cells was monitored using lipophilic probe JC-1 followed by flow cytometric detection. SH-SY5Y cells were plated at a density of 1 × 10^6^ cells per 6 cm dish. Shortly, after 24 h incubation in the presence of tested compounds, cells were detached with Accutase and stained using BD™ MitoScreen (JC-1) Kit according to the manufacturer’s protocol. JC-1 accumulates within intact mitochondria to form multimer J-aggregates (red colour; λex = 488 nm, λem = 590 nm) and the colour of the dye changes from red to green (λex = 488 nm, λem=530 nm) due to depolarization of mitochondrial membrane potential. This alteration was analysed on flow cytometer FACS Canto II using FACSDiva software (BD Biosciences, San Jose, CA, USA). The ratio of aggregate (λem = 590 nm) and monomer (λem = 530 nm) fluorescence was used as a measure of mitochondrial depolarization (Δψm).

### 4.10. ATP Levels

Total ATP content of SH-SY5Y cells was determined using a bioluminescence assay (ViaLight^TM^ Plus Kit, Lonza, Basel, Switzerland) according to the instruction of the manufacturer. The kit is based upon the bioluminescent measurement of ATP that is present in all metabolically active cells. The bioluminescent method utilizes an enzyme, luciferase, which catalyses the formation of light from ATP and luciferin. SH-SY5Y cells were plated in 8 replicates into a white 96-well cell culture plate at a density of 1.5 × 10^4^ cells/well. Shortly, after 24 h incubation in the presence of tested compounds, the cells were lysed for 10 min at RT and the AMR plus reagent was added. After 2 min incubation at RT, the bioluminescence was measured using fluorescence spectrophotometer (FLUOstar Omega; BMG LABTECH, Ortenberg, Germany).

### 4.11. Mitochondrial Redox Environment

To investigate changes in mitochondrial redox environment, SH-SY5Y cells were transfected with a plasmid coding for a redox-sensitive green fluorescent protein with a mitochondrial targeting sequence (pRA306 in pEGFP-N1). In an oxidized environment the absorption increases at short wavelengths (375 nm) at the expense of absorption at longer wavelengths (500 nm). The fluorescence ratio indicates oxidation/reduction as described previously by [139]. SH-SY5Y cells were transfected using electroporation (Neon Transfection System) in 100μl volume containing 1.4 × 10^6^ cells and 20μg DNA, at manufacturer’s SH-SY5Y-optimized pulse parameters (Thermo Fisher Scientific). Cells were plated in 4 replicates onto 96-well plates at a density of 1.5 × 10^4^ cells/well in standard culture medium less antibiotics and kept overnight at 37 °C in 5% CO_2_. After 24 h treatment with oligomeric α-Syn, cells were washed twice with PBS and placed in a Hank’s buffer. The ratio 375 nm/500 nm was measured using multiplate reader Infinite M1000 PRO (TECAN). An increase of the ratio indicates a more oxidized environment. 

### 4.12. Western Blot Analysis 

The cells were washed twice with ice-cold PBS and lysed in Cell Lysis Buffer (1x). Protein levels were determined using the Bradford method, and then the samples were mixed with Laemmli buffer and denatured at 95°C for 5 min. Equal amounts of proteins were separated on SDS/PAGE gels. All proteins were transferred to nitrocellulose membranes at 100 V. Membranes were washed for 5 min in TBS-Tween buffer (0.1% TBST) (100 mM Tris-buffered saline, 140 mM NaCl, and 0.1% Tween 20; pH 7.6) and the nonspecific bindings were blocked for 1 h at RT with 5% BSA in 0.1% TBST or with 5% nonfat milk solution in 0.1% TBST. Immunodetection was performed overnight at 4 °C using rabbit antiparkin (1:500; Cell Signaling), rabbit anti-AMPK (1:1000, Cell Signalling), rabbit anti-p-AMPK (1:1000, Cell Signalling), rabbit anti-Ulk-1 (1:200, Cell Signalling), rabbit anti-p-Ulk-1 (1:200, Cell Signalling), and antimouse total OXPHOS (1:500, Abcam) antibodies. Then, the membranes were washed three times (5 min) in TBST and incubated for 60 min at RT with antirabbit or antimouse secondary antibody (1∶4000) in a 5% nonfat milk/TBST. Antibodies were detected using chemiluminescent Clarity Western ECL Substrate (Bio-Rad Laboratories, Hercules, CA, USA) under standard conditions. Immunolabeling of GAPDH (rabbit anti-GAPDH; 1:40,000; Sigma-Aldrich) for cell lysates was performed as a loading control.

### 4.13. Intracellular Signalling Array

The Intracellular Signaling Protein Array Kit (Chemiluminescent Readout) is a slide-based antibody array founded upon the sandwich immunoassay principle. The array kit allows for the simultaneous detection of 18 important signalling molecules when phosphorylated or cleaved. The cells were washed twice with ice-cold PBS and lysed in Cell Lysis Buffer (1x) supplemented with protease and protein phosphatase inhibitors. Intracellular signalling molecules were detected using a PathScan® Intracellular Signaling Array Kit (Cell Signalling Technology 7323) according to the manufacturer’s protocol procedure. An image of the slide was captured with a digital imaging system. 

### 4.14. Statistical Analysis

The results were expressed as mean values ± S.E.M. Differences between the means were analysed using one-way analysis of variance ANOVA with Bonferroni comparison post hoc test. Statistical significance was accepted at *p* < 0.05. The statistical analyses were performed using Graph Pad Prism version 5.0 (Graph Pad Software, San Diego, CA, USA). 

## Abbreviations

ATP	adenosine 5′-triphosphate
CNS	central nervous system
AD	Alzheimer's disease
PD	Parkinson's disease
ADP	adenosine diphosphate
P2X7R	P2X7 receptor
Panx1	pannexin 1
α-Syn	α-Synuclein
HSP27	heat shock protein 27
SAPK/JNK	stress-activated protein kinases/Jun amino-terminal kinases
MMP	mitochondrial membrane potential
mtROS	mitochondrial reactive oxygen species
ROS	reactive oxygen species
AMPK	AMP-activated protein kinase
ULK1	UNC-51-like kinase 1
LC3-II	microtubule-associated protein 1A light chain 3 II
OXPHOS	oxidative phosphorylation
DAMPs	damage-associated molecular patterns
MAMs	mitochondria-associated endoplasmic reticulum membranes
Panx-1	pannexin-1
OMM	outer mitochondria membrane
PTP	permeability transition pore
